# Retraction note: Investigations of renal function using the level of neutrophil gelatinase-associated lipocalin associated with single-dose of cisplatin during chemotherapy

**DOI:** 10.1186/s13000-016-0559-2

**Published:** 2016-11-02

**Authors:** Omid Maghsoudi, Seyed Hesamoddin Mirjalili, Mojtaba Dolatabadi, Mostafa Fallah Joshaghani, Mojtaba Zarea, Emad Yahaghi, Aram Mokarizadeh

**Affiliations:** 1 Doctor of Veterinary Medicine (DVM), Faculty of Veterinary Medicine, Islamic Azad University, Karaj Branch, Karaj, Iran; 2 Doctor of Veterinary Medicine (DVM), Karaj, Iran; 3 Under graduate of Veterinary Medicine, Faculty of Veterinary Medicine, Islamic Azad University, Karaj Branch, Karaj, Iran; 4 Center for Chemical Biology, Indian Institute of Chemical Technology (iict), Tarnaka, Hyderabad, India; 5 Baqiyatallah University of Medical Sciences, Tehran, Iran; 6 Cellular & Molecular Research Center, Kurdistan University of Medical Sciences, Sanandaj, Iran

## Retraction

The Editor-in-Chief and Publisher have retracted this article [1] because the scientific integrity of the content cannot be guaranteed. An investigation by the Publisher found it to be one of a group of articles we have identified as showing evidence suggestive of attempts to subvert the peer review and publication system to inappropriately obtain or allocate authorship. This article showed evidence of plagiarism (most notably from the articles cited [2–5]) and authorship manipulation.
